# HPLC-DAD-ESI-QTOF-MS/MS qualitative analysis data and HPLC-DAD quantification data of phenolic compounds of grains from five Australian sorghum genotypes

**DOI:** 10.1016/j.dib.2020.106584

**Published:** 2020-11-26

**Authors:** Yun Xiong, Pangzhen Zhang, Robyn Dorothy Warner, Shuibao Shen, Stuart Johnson, Zhongxiang Fang

**Affiliations:** a School of Agriculture and Food, Faculty of Veterinary and Agricultural Sciences, University of Melbourne, Parkville, VIC 3010, Australia; b College of Animal Science and Technology, Guangxi University, Nanning, Guangxi Province, China; c Taiyuan Brand Will Firm Biotechnology Development Co., Ltd, Taiyuan, Shanxi Province, China; d School of Molecular and Life Sciences, Curtin Health Innovation Research Institute, Curtin University, Perth, WA 6845, Australia

**Keywords:** Sorghum, Phenolic compounds, HPLC-DAD-ESI-QTOF-MS/MS, Comprehensive profile, Quantification

## Abstract

Sorghum (*Sorghum bicolor*) grain is a rich source of bioactive phenolic compounds and understanding the phenolic profile of different sorghum genotypes is an important step towards the selection of the most appropriate genotype for industrial applications. The free and bound phenolic compounds of sorghum bran and kernel fractions from five Australian-grown sorghum genotypes (1 white, 2 red, 1 brown and 1 black coloured grain) were identified/tentatively identified by HPLC-DAD-ESI-QTOF-MS/MS and quantified/semi-quantified by HPLC-DAD. Firstly, MS chromatograms of sorghum samples and standards and the MS/MS spectra of individual detected compounds and standards are presented. Then quantification data of these compounds is provided. This dataset is supplementary to the research paper “Comprehensive profiling of phenolic compounds by HPLC-DAD-ESI-QTOF-MS/MS to reveal their location and form of presence in different sorghum grain genotypes” [1].

**Specifications Table**

**Table:** 

Subject	Agricultural and Biological Sciences (General)
Specific subject area	Mass spectrometry, phytochemistry
Type of data	Table, Fig.
How data were acquired	The mass spectrometry data was obtained from Agilent 6520I Accurate-Mass Q-TOF LC/MS coupled to an Agilent 1200 series HPLC system (Agilent Technologies, USA). The quantification data was obtained from Agilent 1260 series HPLC-DAD (Agilent Technologies, USA). A Synergi Hydro-RP 80A LC column (4 µm, 250 × 4.6 mm) protected by an AQ C18 guard column (4.0 × 3.0 mm) (Phenomenex, Australia) was used.
Data format	Raw, analysed
Parameters for data collection	MS: negative mode via a dual electrospray ionisation source (ESI), drying gas N_2_, temperature 325 °C, gas flow 9 L/min, nebuliser 45 psi; capillary voltage 3500 V, fragmentor 175 V, MS scan range 90-1000 m/z. MS/MS: auto mode, scan range 90-850 m/z, collision energy 15-30 eV.
Description of data collection	The MS chromatograms of sorghum samples and standards, and the MS/MS spectra of phenolic compounds and standards were obtained by MassHunter Qualitative software (Agilent Technologies, USA). The quantification data was analysed by Agilent OpenLAB workstation (Agilent Technologies, USA).
Data source location	Liberty, Mr-Buster, Nuseed Cracka sorghum grains were obtained from Nuseed Australia (Toowoomba, QLD, Australia) in 2019. IS131C and Shawaya Short Black 1 sorghum grains were obtained from the experiment filed of Bentley campus of Curtin University, grown January to April 2019 (Bentley, WA, Australia).
Data accessibility	With the article
Related research article	Xiong Y, Zhang P, Warner RD, Shen S, Johnson S, Fang Z. Comprehensive profiling of phenolic compounds by HPLC-DAD-ESI-QTOF-MS/MS to reveal their location and form of presence in different sorghum grain genotypes. Food Research International. 127 (2020) 109671. DOI: 10.1016/j.foodres.2020.109671

## Value of the Data

• The MS chromatogram and MS/MS spectra data can be used as a reference, and serve as a benchmark, for the identification of phenolic compounds in sorghum grains; the quantification data provide useful information for the evaluation and estimation of individual or group of phenolic contents in sorghum grain materials.
• The qualitative and quantitative data provide valuable information/reference to researchers from various sectors (agricultural, food and pharmaceutical) for the analysis and comparison of phenolic compounds in sorghum as well as in other cereal grains or plant materials.
• The data provide a comprehensive understanding of the sorghum phenolic profile, which provides useful insights into sorghum material selection and processing design to help tailor specific industrial food or drug applications of sorghum.

## 1 Data Description

**Fig. 1:**
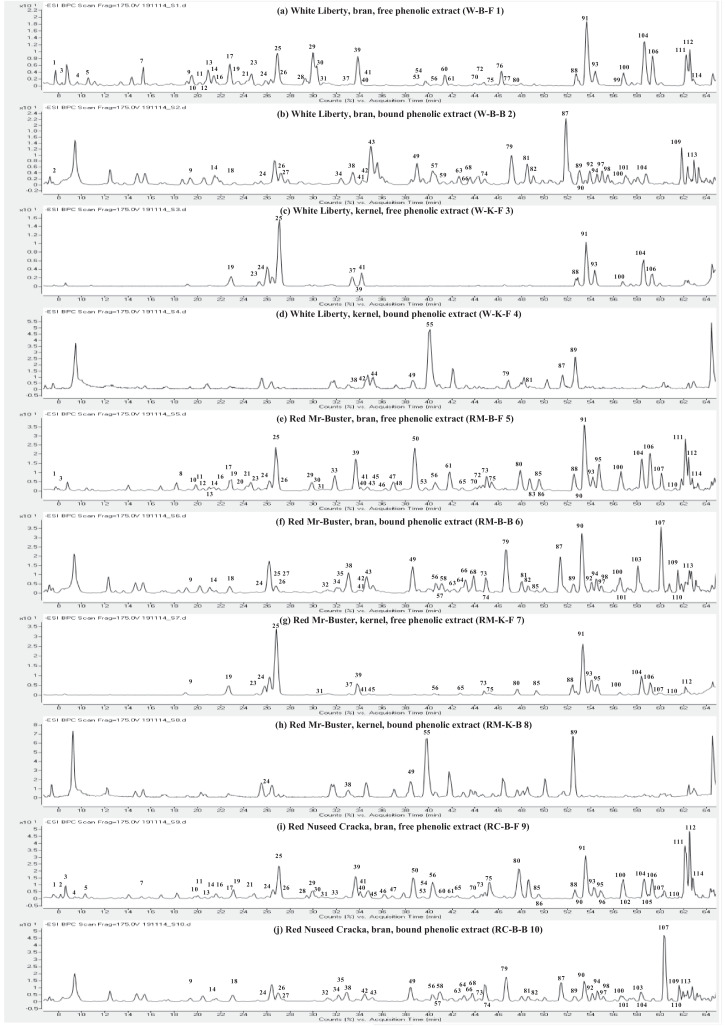

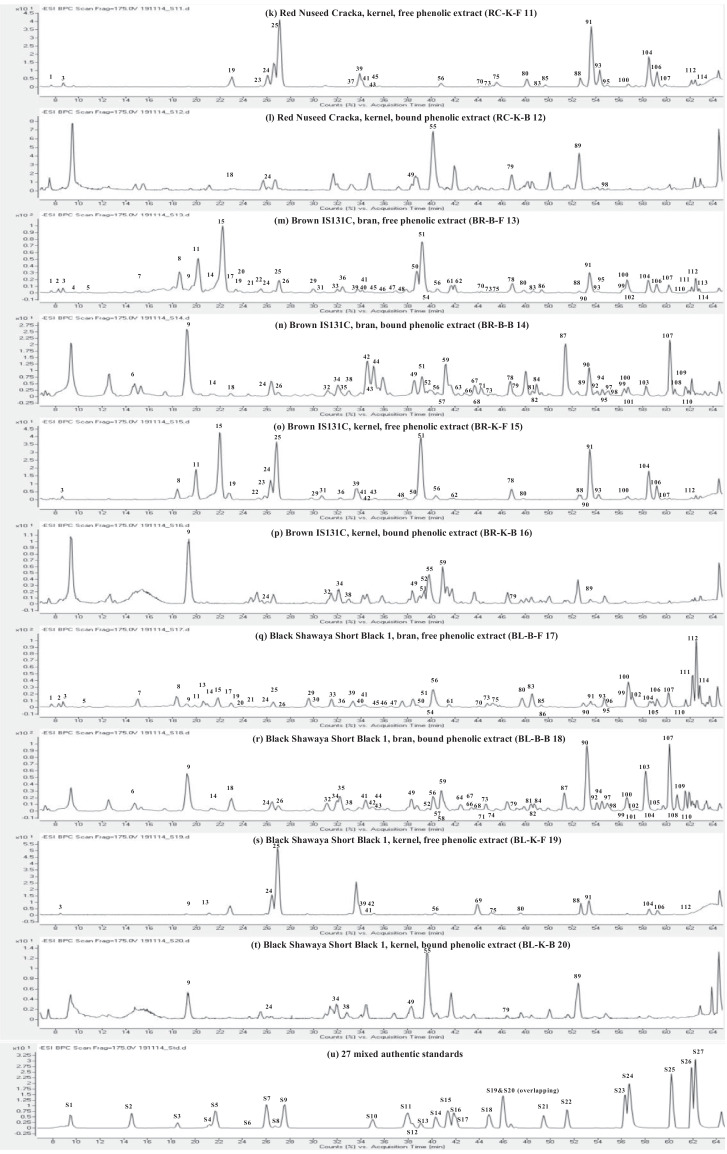
MS chromatograms of 20 sorghum samples (a-t) and mixed standards (u). Peak numbers 1-114 referring to Table 1 in Reference [1] and Fig. 3; peak numbers S1-27 referring to Fig. 2.

**Fig. 2:**
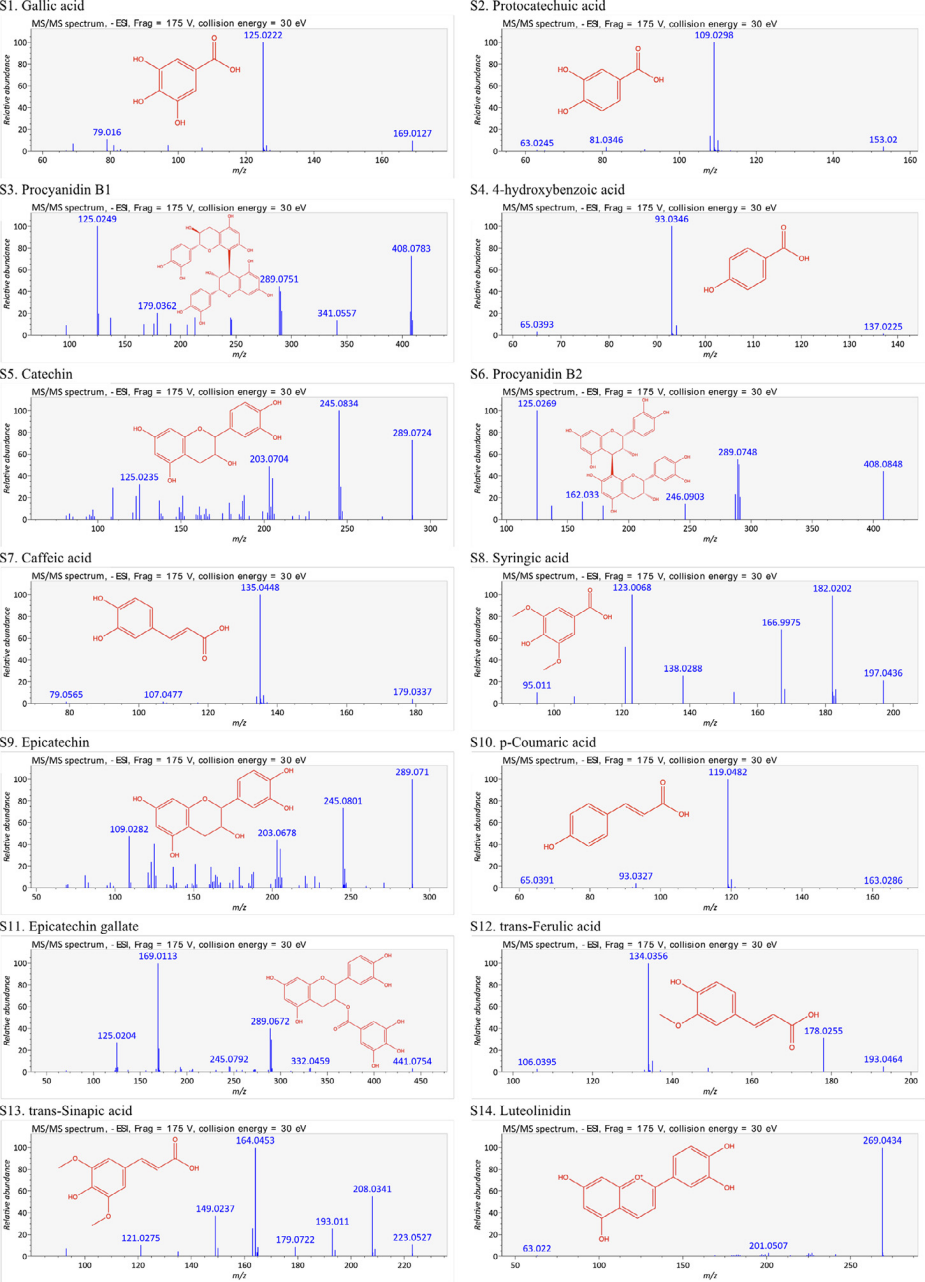

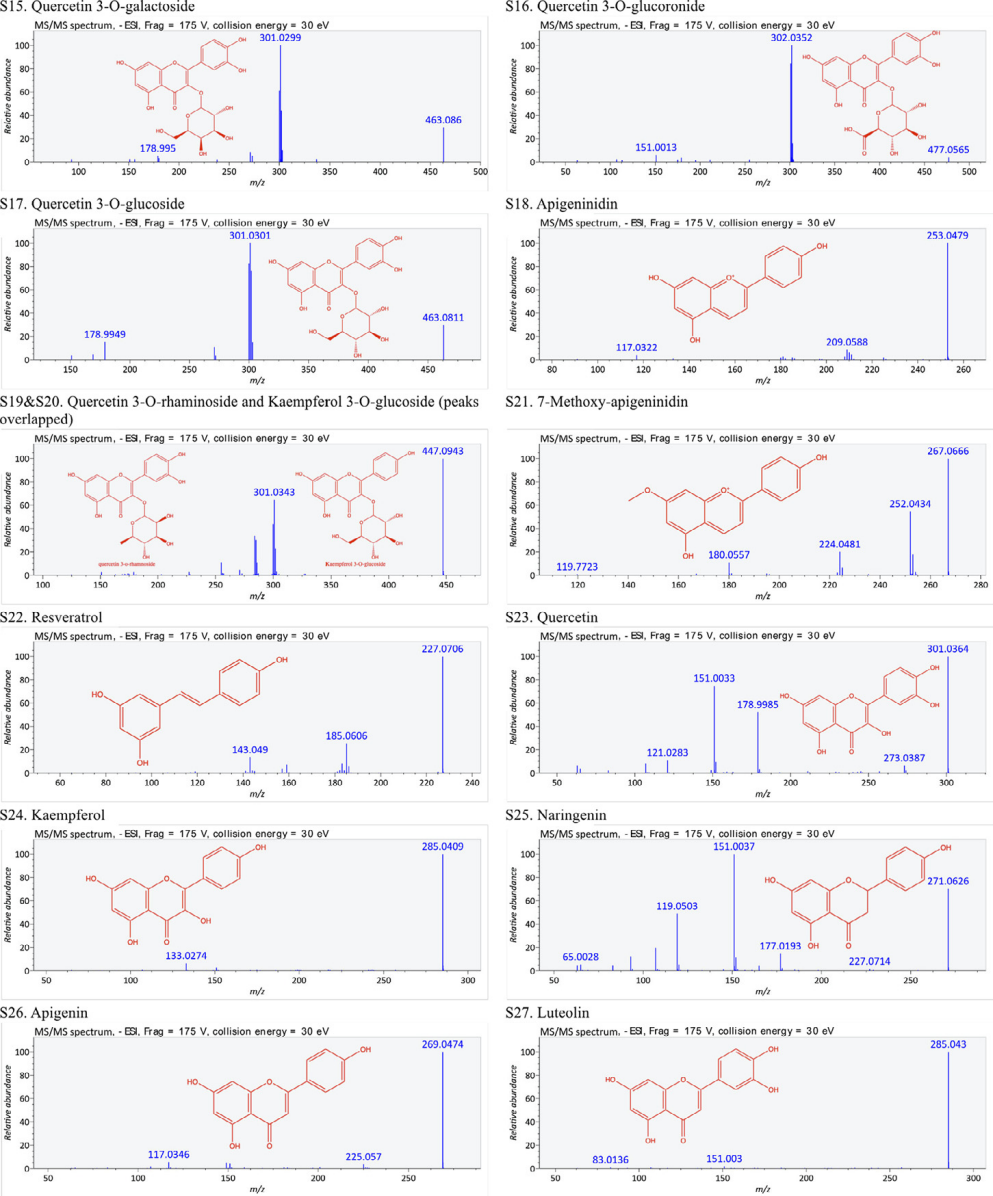
The MS/MS spectrum and structure of 27 standards. Standard peak numbers S1-27 are shown in Fig. 1.

**Fig. 3:**
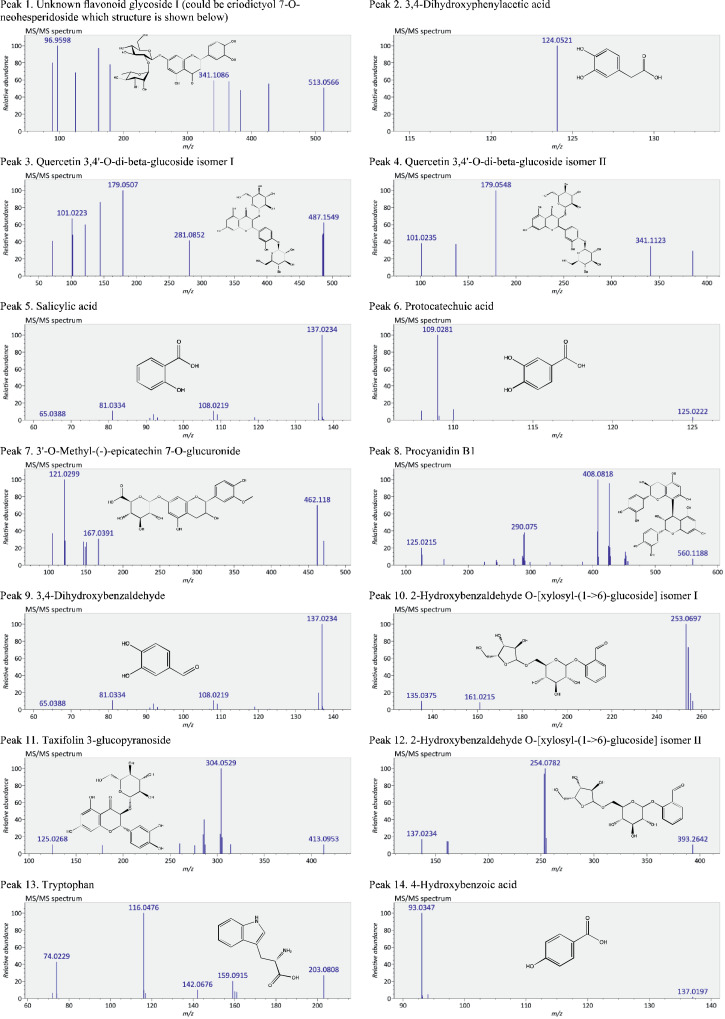

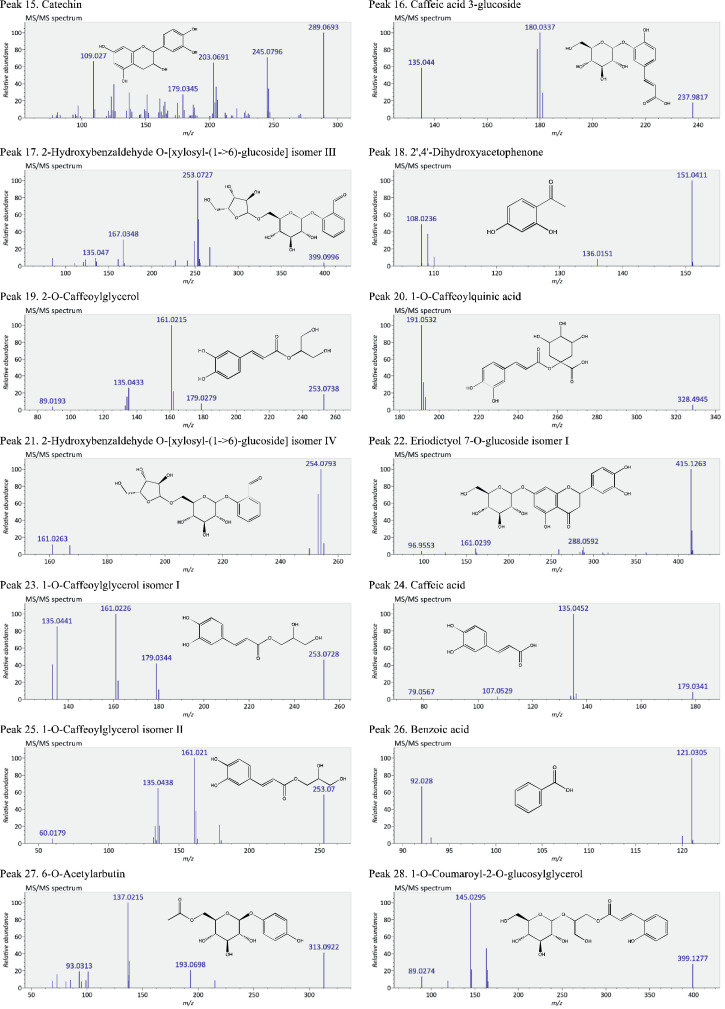

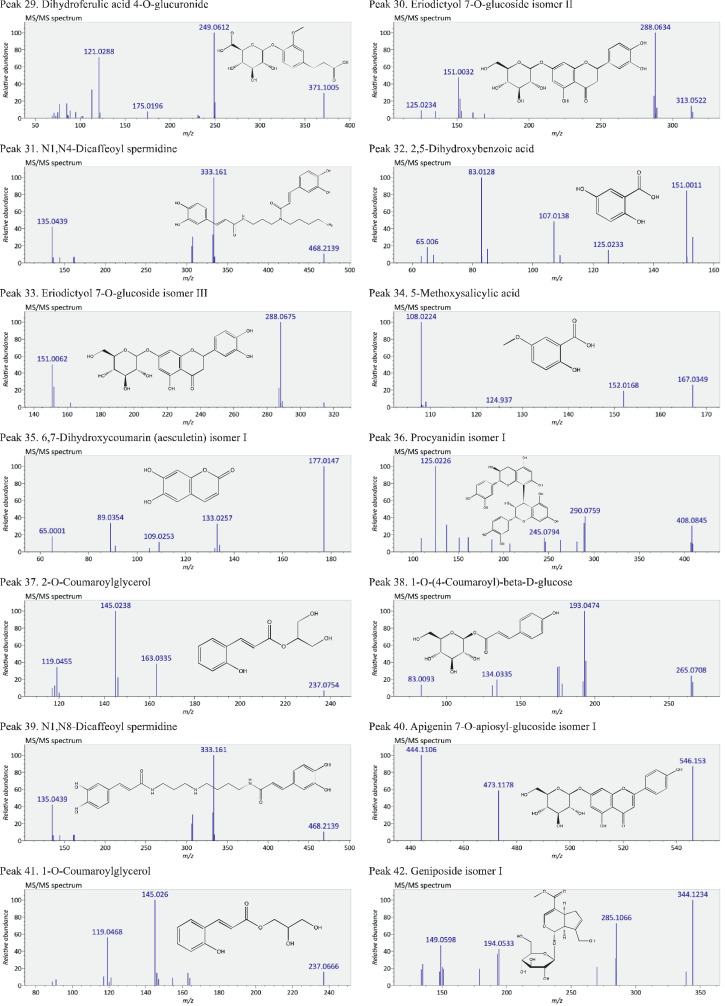

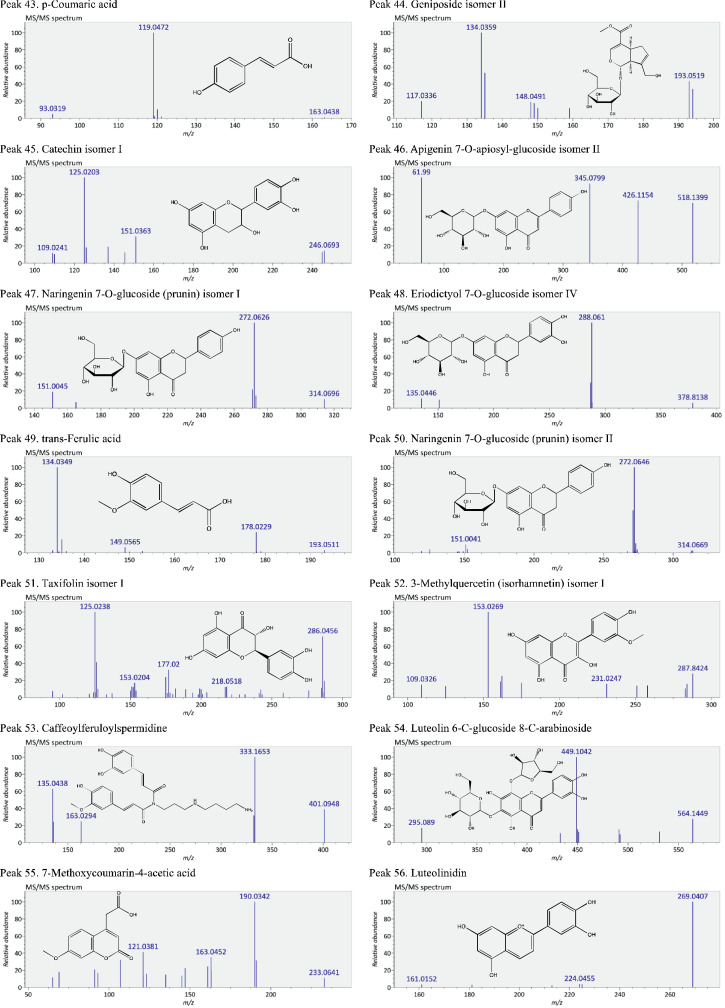

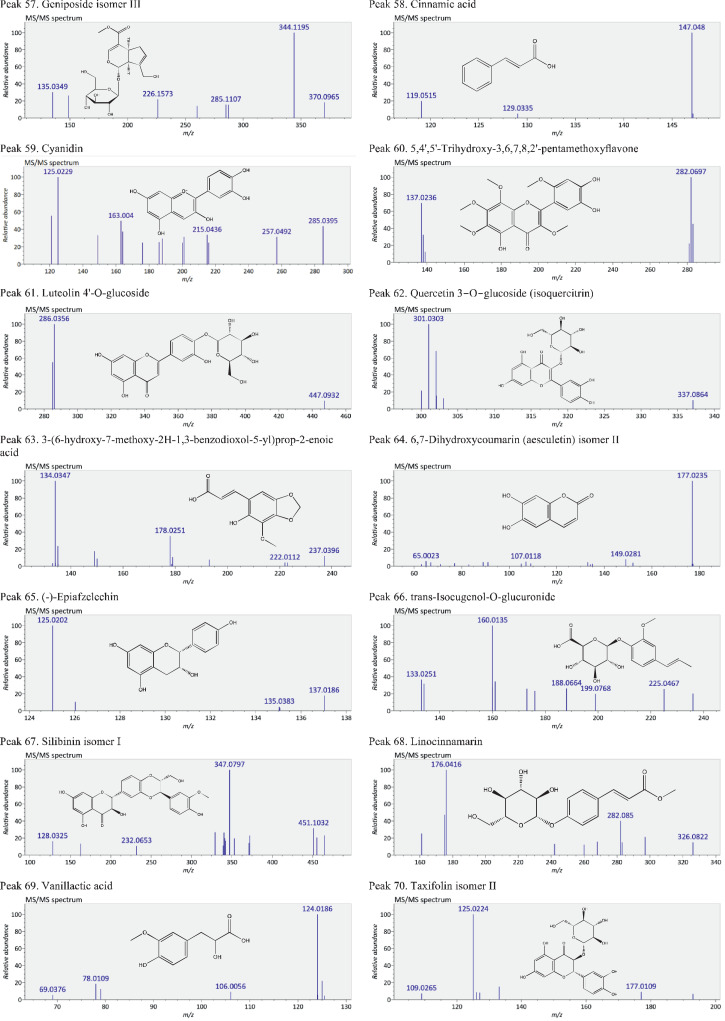

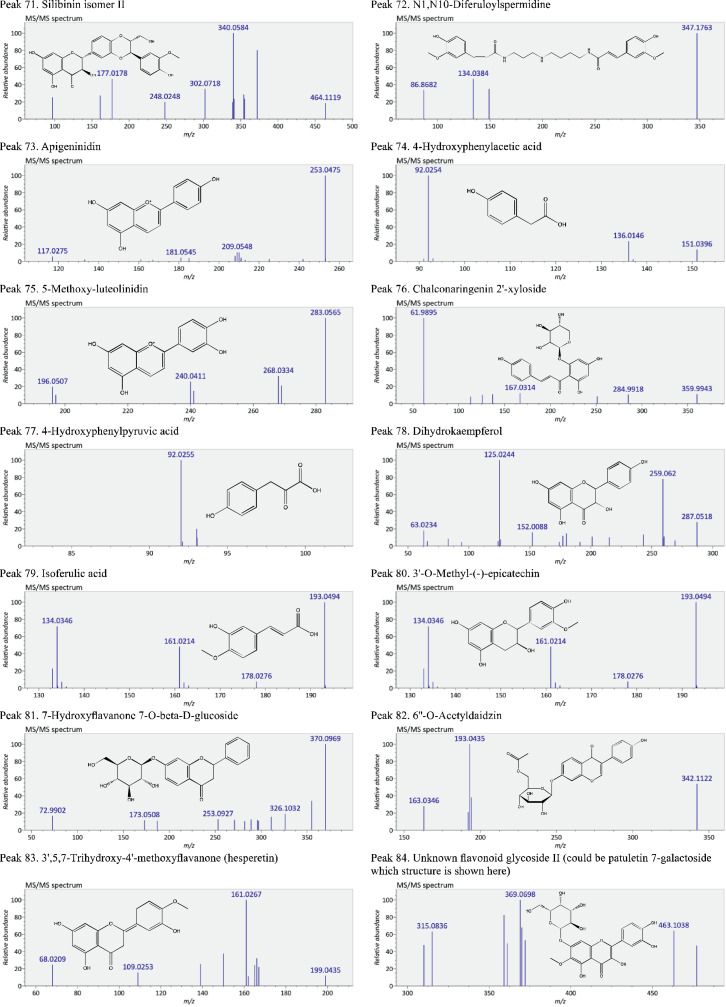

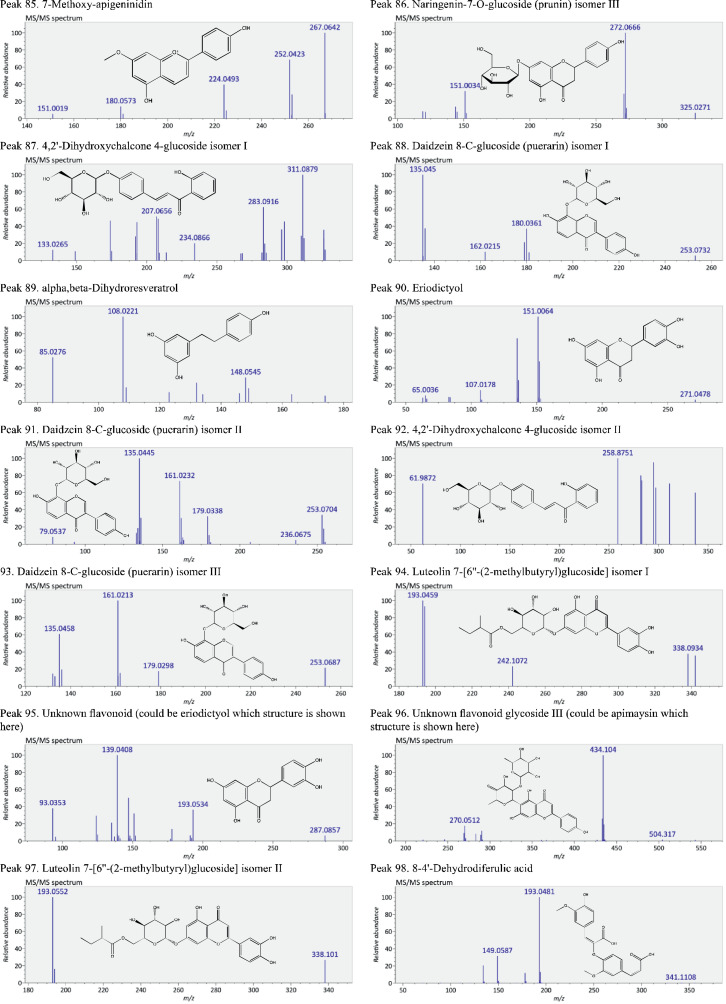

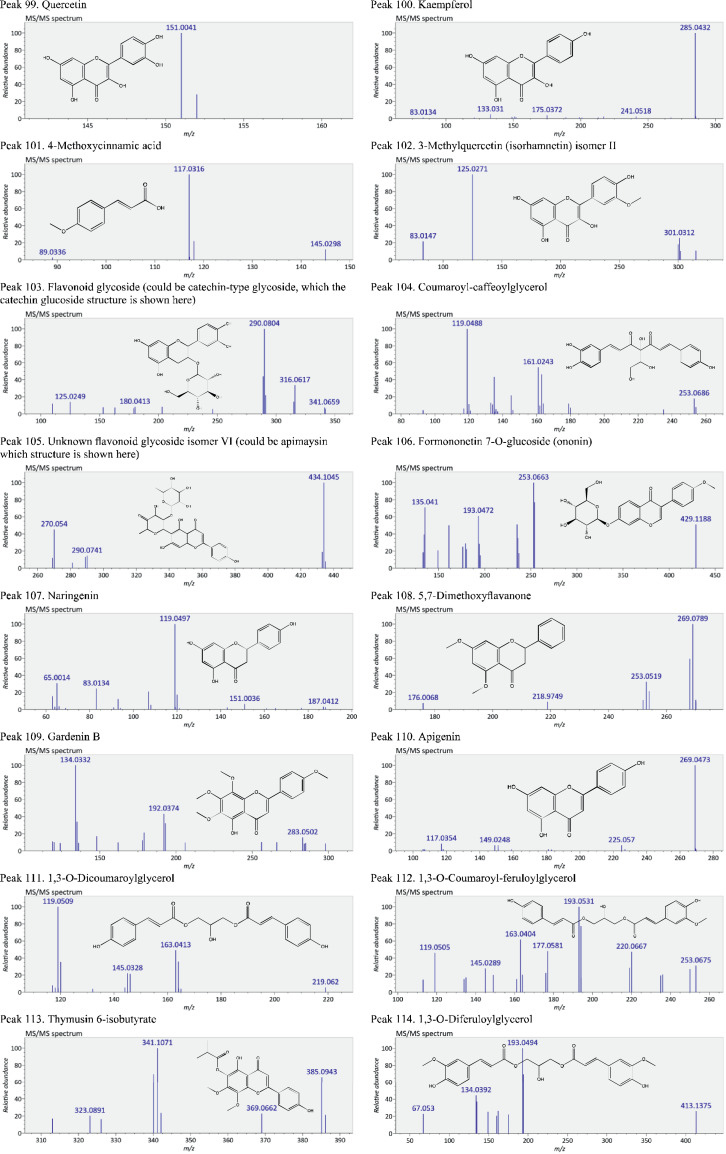
The MS/MS spectrum and structure of 114 identified or tentatively identified compounds in sorghum samples. Compound peak numbers 1-114 are shown in Fig. 1.

This present dataset provides supplementary information to our work submitted to Reference [1]. The MS chromatograms of 20 sorghum samples (i.e. free and bound phenolic extracts of bran and kernel fractions from 5 sorghum grain genotypes) and a standard sample of 27 mixed phenolic standards are provided in Fig. 1. Data in Table 1 presents the 27 phenolic standards used for identification and their retention time, precursor ion and presence in the tested sorghum samples. The MS/MS spectrum and structure of 27 standards used are provided in Fig. 2. The MS/MS spectrum and structure of 114 identified or tentatively identified compounds in sorghum samples are provided in Fig. 3. Data in Table 2 were the calibration and method validation parameters for the quantification of phenolic compounds. Data in Table 3 presents the concentration of phenolic compounds and the standards used for their quantification or semi-quantification.

**Table 1:** Authentic standards used for identification.

Peak number	Standards	Retention time (min)	[M-H]- (m/z)	Detected in sorghum samples
S1	Gallic acid	9.456	169.0121	ND
S2	Protocatechuic acid	14.669	153.0203	Yes
S3	Procyanidin B1	18.480	577.1430	Yes
S4	4-hydroxybenzoic acid	21.147	137.0248	Yes
S5	Catechin	21.680	289.0748	Yes
S6	Procyanidin B2	24.632	577.1456	ND
S7	Caffeic acid	25.987	179.0342	Yes
S8	Syringic acid	26.725	197.0441	ND
S9	Epicatechin	17.574	289.0716	ND
S10	*p-*coumaric acid	34.927	163.0385	Yes
S11	Epicatechin gallate	37.935	441.0833	ND
S12	trans-Ferulic acid	38.300	193.0485	Yes
S13	trans-Sinapic acid	39.072	223.0597	ND
S14	Luteolinidin	40.247	269.0443	Yes
S15	Quercetin 3*-O-*galactoside	41.326	463.0871	ND
S16	Quercetin 3*-O-*glucoronide	41.868	477.0672	ND
S17	Quercetin 3*-O-*glucoside	42.046	463.0890	Yes
S18	Apigeninidin	44.727	253.0497	Yes
S19	Quercetin 3*-O-*rhaminoside	45.994	447.0931	ND
S20	Kaempferol 3*-O-*glucoside	45.994	447.0931	ND
S21	7-Methoxyapigeninidin	49.340	267.0669	Yes
S22	Resveratrol	51.444	227.0720	ND
S23	Quercetin	56.240	301.0376	Yes
S24	Kaempferol	56.587	285.0421	Yes
S25	Naringenin	60.160	271.0628	Yes
S26	Apigenin	61.847	269.0477	Yes
S27	Luteolin	62.239	285.0429	ND

Standard peak numbers S1-27 are shown in Fig. 1 (u).

ND = not detected

**Table 2:** HPLC-DAD method validation parameters for the quantification of phenolic compounds.

								Repeatability	

Peak number	Standards	Quantification λ (nm)	Calibration curve	R^2^	Linearity range (µg/mL)	LOD (µg/mL)	LOQ (µg/mL)	Conc. (µg/mL)	RSD% (n=3)
S2	Protocatechuic acid	280	y = 2.7382x + 0.8849	0.9997	0.4-100	0.28	0.84	50	3.84
								25	4.03
								12.5	1.78
S3	Procyanidin B1	280	y = 1.0122x - 0.0470	0.9986	0.7-40	0.37	1.12	40	3.45
								20	3.21
								10	1.49
S4	4-hydroxybenzoic acid	280	y = 2.6027x + 0.2256	0.9996	0.4-100	0.31	0.93	50	3.78
								25	4.11
								12.5	5.91
S5	Catechin	280	y = 1.1088x + 1.0751	0.9999	0.8-200	0.23	0.71	200	0.24
								100	1.72
								50	2.72
S7	Caffeic acid	320	y = 5.0965x - 0.3624	0.9998	0.4-100	0.31	0.93	50	4.53
								25	3.45
								12.5	2.88
S10	*p*-coumaric acid	320	y = 7.7790x + 0.2580	0.9999	0.4-100	0.19	0.57	50	4.04
								25	3.57
								12.5	2.19
S12	trans-Ferulic acid	320	y = 5.2207x + 2.5300	0.9999	0.8-200	0.27	0.82	200	0.06
								100	2.75
								50	1.79
S14	Luteolinidin	485	y = 2.9103x - 2.2840	0.9999	0.8-200	0.36	1.09	200	0.11
								100	3.28
								50	1.66
S17	Quercetin 3*-O-*glucoside	370	y = 1.5905x - 0.1083	0.9983	0.4-100	0.67	2.02	50	4.60
								25	7.30
								12.5	4.82
S18	Apigeninidin	485	y = 5.0106x +1.0940	0.9999	0.8-200	0.20	0.60	200	0.07
								100	3.00
								50	1.93
S21	7-Methoxyapigeninidin	485	y = 2.2525x - 0.2609	0.9999	0.4-100	0.12	0.35	50	4.58
								25	3.52
								12.5	4.79
S23	Quercetin	370	y = 0.6596x - 0.4096	0.9980	0.4-100	0.73	2.20	50	4.07
								25	8.95
								12.5	1.78
S24	Kaempferol	370	y = 1.2170x - 0.0813	0.9995	0.4-100	0.35	1.06	50	2.17
								25	1.29
								12.5	6.29
S25	Naringenin	280	y = 4.7151x + 0.1801	0.9999	0.4-100	0.10	0.32	50	3.96
								25	3.71
								12.5	2.06
S26	Apigenin	340	y = 4.8070x - 1.6658	0.9998	0.4-100	0.24	0.73	50	4.09
								25	2.74
								12.5	3.10

Standard peak numbers S1-27 are shown in Fig. 1.

LOD = limits of detection; LOQ = limits of quantification; RSD = relative standard deviation.

**Table 3:** Quantification of sorghum phenolic compounds by HPLC-DAD.


				Concentration of quantified compounds (µg/g)																			

Class	Name of Compound	Peak No	Standard used for quantification	W-B-F 1	W-B-B 2	W-K-F 3	W-K-B 4	RM-B-F 5	RM-B-B 6	RM-K-F 7	RM-K-B 8	RC-B-F 9	RC-B-B 10	RC-K-F 11	RC-K-B 12	BR-B-F 13	BR-B-B 14	BR-K-F 15	BR-K-B 16	BL-B-F 17	BL-B-B 18	BL-K-F 19	BL-K-B 20

Phenolic acid																							

Hydroxybenzoic acid (B)	Salicylic acid	5	4-Hydroxybenzoic acid	41.93±2.80	0	0	0	11.07±1.69	0	0	0	27.61±0.71	0	0	0	40.29±0.89	0	0	0	109.98±3.11	0	0	0
	Protocatechuic Acid	6	Protocatechuic Acid	0	0	0	0	0	0	0	0	0	0	0	0	0	22.83±0.60	0	0	0	26.10±3.45	0	0
	4-Hydroxybenzoic acid	14	4-Hydroxybenzoic acid	121.01±2.32	91.69±1.74	0	0	59.32±1.03	36.44±1.78	0	0	74.57±1.54	48.85±2.20	0	0	BDT	18.20±3.07	0	0	BDT	36.67±2.40	0	0
	Benzoic acid	26	4-Hydroxybenzoic acid	BDT	UQ	0	0	BDT	UQ	0	0	BDT	UQ	0	0	BDT	UQ	0	0	BDT	64.97±6.00	0	0
	2,5-Dihydroxybenzoic acid	32	4-Hydroxybenzoic acid	0	0	0	0	0	BDT	0	0	0	BDT	0	0	0	14.03±3.02	0	BDT	0	24.12±3.30	0	0

Hydroxycinnamic acid (C)	Caffeic acid 3-glucoside	16	Caffeic Acid	BDT	0	0	0	3.82±0.19	0	0	0	4.31±0.95	0	0	0	0	0	0	0	0	0	0	0
	2*-O-*Caffeoylglycerol	19	Caffeic Acid	BDT	0	4.63±0.55	0	BDT	0	7.84±0.16	0	BDT	0	6.05±0.55	0	BDT	0	5.14±0.70	0	BDT	0	0	0
	1*-O-*Caffeoylquinic acid	20	Caffeic Acid	0	0	0	0	BDT	0	0	0	0	0	0	0	22.99±0.91	0	0	0	9.84±2.98	0	0	0
	1*-O-*Caffeoylglycerol I	23	Caffeic Acid	BDT	0	10.21±2.29	0	BDT	0	BDT	0	0	0	BDT	0	0	0	BDT	0	0	0	0	0
	Caffeic Acid	24	Caffeic Acid	10.68±1.06	UQ	BDT	0	19.2±1.07	UQ	2.17±1.80	UQ	19.53±0.73	UQ	32.75±1.74	UQ	23.55±3.28	UQ	7.93±0.71	UQ	BDT	78.12±1.87	13.24±0.23	0
	1*-O-*Caffeoylglycerol II	25	Caffeic Acid	55.80±1.56	0	21.82±1.48	0	45.94±1.77	BDT	42.9±1.31	0	40.73±0.69	0	35.18±0.82	0	30.03±2.07	0	21.81±0.46	0	68.51±3.74	BDT	19.24±0.88	0
	1*-O-*Coumaroyl-2*-O-*glucosylglycerol	28	*p-*Coumaric Acid	6.12±0.17	0	0	0	0	0	0	0	BDT	0	0	0	0	0	0	0	0	0	0	0
	Dihydroferulic acid 4*-O-*glucuronide	29	trans-Ferulic Acid	1.68±0.46	0	0	0	4.64±0.29	0	0	0	BDT	0	0	0	17.41±0.94	0	0.17±0.23	0	15.56±0.51	0	0	0
	N1,N4-Dicaffeoyl spermidine	31	Caffeic Acid	BDT	0	0	0	BDT	0	BDT	0	BDT	0	0	0	BDT	0	4.85±0.24	0	0	0	0	0
	2*-O-*Coumaroylglycerol	37	*p-*Coumaric Acid	BDT	0	2.34±0.73	0	0	0	1.48±0.27	0	0	0	1.27±0.05	0	0	0	0	0	0	0	0	0
	1*-O-*(4-Coumaroyl)-beta-D-glucose	38	*p-*Coumaric Acid	0	11.05±0.92	0	BDT	0	24.47±2.96	0	2.35±0.77	0	11.85±0.67	0	0	0	9.05±2.11	0	8.28±3.14	0	7.46±0.70	0	BDT
	N1,N8-Dicaffeoyl spermidine	39	Caffeic Acid	49.14±0.88	0	BDT	0	73.37±1.71	0	20.62±1.07	0	46.61±1.31	0	20.09±1.53	0	45.89±4.66	0	18.91±0.70	0	36.64±2.78	0	8.40±1.24	0
	1*-O-*Coumaroylglycerol	41	*p-*Coumaric Acid	BDT	BDT	6.41±0.71	0	BDT	BDT	BDT	0	BDT	BDT	BDT	0	BDT	0	BDT	0	BDT	BDT	4.04±0.29	0
	*p-*Coumaric Acid	43	*p-*Coumaric Acid	0	91.67±2.37	0	0	8.98±0.94	66.21±4.01	0	0	0	52.66±2.42	BDT	0	0	BDT	2.88±0.16	0	0	BDT	0	0
	trans-Ferulic Acid	49	trans-Ferulic Acid	0	273.82±2.42	0	17.68±1.41	0	391.41±9.60	0	30.03±2.17	0	256.20±3.13	0	27.14±0.79	0	80.71±6.04	0	27.74±0.98	0	234.19±5.61	0	22.65±1.72
	Caffeoylferuloylspermidine	53	Caffeic Acid	BDT	0	0	0	22.89±0.87	0	0	0	BDT	0	0	0	0	0	0	0	0	0	0	0
	Cinnamic acid	58	trans-Ferulic Acid	0	0	0	0	0	5.90±1.02	0	0	0	8.42±0.96	0	0	0	0	0	0	0	BDT	0	0
	3-(6-hydroxy-7-methoxy-2H-1,3-benzodioxol-5-yl)prop-2-enoic acid	63	trans-Ferulic Acid	0	3.12±0.83	0	0	0	BDT	0	0	0	2.34±0.29	0	0	0	BDT	0	0	0	0	0	0
	Linocinnamarin	68	trans-Ferulic Acid	0	20.02±1.11	0	0	0	15.06±4.80	0	0	0	9.17±1.08	0	0	0	BDT	0	0	0	BDT	0	0
	N1,N10-Diferuloylspermidine	72	trans-Ferulic Acid	1.57±0.37		0	0	BDT	0	0	0	0	0	0	0	0	0	0	0	0	0	0	0
	Isoferulic acid	79	trans-Ferulic Acid	0	13.96±1.44	0	BDT	0	28.17±3.10	0	0	0	20.35±1.30	0	0.28±0.17	0	6.47±1.11	0	BDT	0	7.95±1.34	0	BDT
	8-4′-Dehydrodiferulic acid	98	trans-Ferulic Acid	0	43.62±1.25	0	0	0	BDT	0	0	0	25.55±0.58	0	BDT	0	20.27±0.34	0	0	0	61.82±1.32	0	0
	4-Methoxycinnamic acid	101	trans-Ferulic Acid	0	19.32±0.53	0	0	0	7.90±1.29	0	0	0	BDT	0	0	0	BDT	0	0	0	BDT	0	0
	Coumaroyl-caffeoylglycerol	104	*p-*Coumaric Acid	12.63±1.00	BDT	3.39±0.26	0	0.85±2.04	0	6.61±0.22	0	14.22±0.77	BDT	9.71±0.58	0	12.96±0.70	0	6.81±0.11	0	4.74±0.71	BDT	1.64±0.19	0
	1,3*-O-*Dicoumaroylglycerol	111	*p-*Coumaric Acid	6.49±0.34	0	0	0	13.35±0.92	0		0	34.25±0.18	0	0	0	6.06±0.97	0	0	0	44.51±2.40	0	0	0
	1,3*-O-*Coumaroyl-feruloylglycerol	112	*p-*Coumaric Acid	8.63±0.61	0	0	0	18.45±0.48	0	BDT	0	54.06±0.48	0	BDT	0	16.84±0.77	0	1.59±0.18	0	90.37±1.74	0	BDT	0
	1,3*-O-*Diferuloylglycerol	114	trans-Ferulic Acid	1.42±0.23	0	0	0	3.80±0.93	0	0	0	14.93±0.40	0	BDT	0	4.93±0.66	0	0	0	31.09±0.89	0	0	0
Flavonoids																							

3-Deoxyanthocyanidin (3DA)	Luteolinidin	56	Luteolinidin	18.18±0.20	0	0	0	104.37±2.83	54.29±2.19	8.26±0.44	0	61.68±2.35	36.92±1.86	9.45±0.61	0	37.57±1.62	13.62±0.97	9.89±0.52	0	171.59±4.43	76.35±1.27	7.99±0.31	0
	Apigeninidin	73	Apigeninidin	0	0	0	0	242.08±7.39	30.35±2.24	3.84±0.45	0	244.77±2.45	52.85±2.73	2.05±0.41	0	372.51±5.41	BDT	0	0	38.88±4.38	22.37±1.29	0	0
	5-Methoxy-luteolinidin	75	Luteolinidin	BDT	0	0	0	25.22±4.06	0	6.67±0.70	0	18.61±1.58	BDT	8.15±0.66	0	48.92±1.90	0	0	0	28.35±3.21	0	5.23±0.18	0
	7-Methoxy-apigeninidin	85	7-Methoxy-apigeninidin	0	0	0	0	53.02±1.52	BDT	8.25±0.87	0	37.57±1.34	0	3.87±0.74	0	0	0	0	0	BDT	0	0	0
Anthocyanidin (A)	Cyanidin	59	Luteolinidin	0	BDT	0	0	0	0	0	0	0	0	0	0	0	192.71±24.06	0	56.01±4.12	0	236.57±6.86	0	0

Flavan-3-ol (F3OL)	3′*-O-*Methyl-(-)-epicatechin 7*-O-*glucuronide	7	Catechin	1.15±0.29	0	0	0	0	0	0	0	BDT	0	0	0	13.40±1.19	0	0	0	11.45±1.93	0	0	0
	Catechin	15	Catechin	0	0	0	0	0	0	0	0	0	0	0	0	1210.70±24.39	0	106.37±4.24	0	116.11±2.73	0	0	0
	Catechin I	45	Catechin	0	0	0	0	BDT	0	10.24±0.80	0	BDT	0	13.23±1.44	0	BDT	0	0	0	BDT	0	0	0
	(-)-Epiafzelechin	65	Catechin	0	0	0	0	31.47±0.99	0	7.23±0.53	0	BDT	0	0	0	0	0	0	0	0	0	0	0
	3′*-O-*Methyl-(-)-epicatechin	80	Catechin	BDT	0	0	0	217.54±4.19	0	7.87±0.76	0	80.15±2.86	0	5.53±0.70	0	69.68±6.95	0	BDT	0	95.79±5.01	0	BDT	0
	Flavonoid glycoside (catechin or catechin type)	103	Catechin	0	0	0	0	0	936.08±27.12	0	0	0	783.84±20.40	0	0	0	403.78±34.04	0	0	0	BDT	0	0
*(continued on next page)*

**Table:** **Table 3** (continued)


				Concentration of quantified compounds (µg/g)																			

Class	Name of Compound	Peak No	Standard used for quantification	W-B-F 1	W-B-B 2	W-K-F 3	W-K-B 4	RM-B-F 5	RM-B-B 6	RM-K-F 7	RM-K-B 8	RC-B-F 9	RC-B-B 10	RC-K-F 11	RC-K-B 12	BR-B-F 13	BR-B-B 14	BR-K-F 15	BR-K-B 16	BL-B-F 17	BL-B-B 18	BL-K-F 19	BL-K-B 20

Flavanone (FN)	Unknown flavonoid glycoside I (eriodictyol 7*-O-*neohesperidoside or eriodictyol type)	1	Naringenin	4.88±0.57	0	0	0	0.96±0.08	0	0	0	1.42±0.28	0	1.85±0.23	0	2.06±0.39	0	0	0	2.87±0.36	0	0	0
	Eriodictyol 7*-O-*glucoside I	22	Naringenin	0	0	0	0	0	0	0	0	0	0	0	0	78.53±2.29	0	BDT	0	0	0	0	0
	Eriodictyol 7*-O-*glucoside II	30	Naringenin	10.65±1.07	0	0	0	BDT	0	0	0	BDT	0	0	0	0	0	0	0	BDT	0	0	0
	Eriodictyol 7*-O-*glucoside III	33	Naringenin	0	0	0	0	44.95±2.42	0	0	0	4.39±0.23	0	0	0	27.93±1.67	0	0	0	61.76±3.03	0	0	0
	Naringenin 7*-O-*glucoside (prunin) I	47	Naringenin	0	0	0	0	1.15±1.81	0	0	0	BDT	0	0	0	25.11±1.08	0	0	0	64.77±3.58	0	0	0
	Eriodictyol 7*-O-*glucoside IV	48	Naringenin	0	0	0	0	BDT	0	0	0	0	0	0	0	BDT	0	1.85±0.26	0	0	0	0	0
	Naringenin 7*-O-*glucoside (prunin) II	50	Naringenin	0	0	0	0	163.70±2.95	0	0	0	45.58±1.06	0	0	0	269.53±4.96	0	0.17±0.38	0	17.73±3.55	0	0	0
	Dihydrokaempferol	78	Naringenin	0	0	0	0	0	0	0	0	0	0	0	0	58.62±2.35	BDT	6.37±0.71	0	0	0	0	0
	7-Hydroxyflavanone 7*-O-*beta-D-glucoside	81	Naringenin	0	6.54±0.47	0	BDT	0	BDT	0	0	0	3.84±0.52	0	0	0	9.23±0.24	0	0	0	8.50±0.87	0	0
	3′,5,7-Trihydroxy-4′-methoxyflavanone (hesperetin)	83	Naringenin	0	0	0	0	BDT	0	0	0	13.91±1.23	0	BDT	0	21.33±3.25	0	0	0	27.10±0.38	0	0	0
	Naringenin-7*-O-*glucoside (prunin) III	86	Naringenin	0	0	0	0	BDT	0	0	0	BDT	0	0	0	BDT	0	0	0	8.51±1.12	0	0	0
	Eriodictyol	90	Naringenin	0	BDT	0	0	BDT	95.62±3.07	0	0	BDT	54.93±3.55	0	0	BDT	42.52±0.24	BDT	0	BDT	171.83±5.37	0	0
	Unknown flavonoid (eriodictyol or flavanone type)	95	Naringenin	0	0	0	0	9.80±0.92	0	BDT	0	12.68±1.46	0	1.87±0.49	0	BDT	BDT	0	0	BDT	0	0	0
	Naringenin	107	Naringenin	0	0	0	0	16.64±0.93	75.39±2.71	BDT	0	BDT	119.49±6.71	BDT	0	19.99±1.84	41.49±2.96	BDT	0	BDT	106.46±2.03	0	0
	5,7-Dimethoxyflavanone	108	Naringenin	0	0	0	0	0	0	0	0	0	0	0	0	0	1.89±0.22	0	0	0	BDT	0	0

Flavone (FO)	Apigenin 7*-O-*apiosyl-glucoside I	40	Apigenin	BDT	0	0	0	BDT	0	0	0	13.19±1.21	0	0	0	23.33±1.83	0	0	0	1.67±1.99	0	0	0
	Apigenin 7*-O-*apiosyl-glucoside II	46	Apigenin	0	0	0	0	BDT	0	0	0	9.90±0.65	0	0	0	BDT	0	0	0	BDT	0	0	0
	Luteolin 6-C-glucoside 8-C-arabinoside	54	Apigenin	7.07±1.61	0	0	0	0	0	0	0	BDT	0	0	0	BDT	0	0	0	BDT	0	0	0
	5,4′,5′-Trihydroxy-3,6,7,8,2′-pentamethoxyflavone	60	Apigenin	6.79±0.61	0	0	0	0	0	0	0	BDT	0	0	0	0	0	0	0	0	0	0	0
	Luteolin 4′*-O-*glucoside	61	Apigenin	BDT	0	0	0	36.86±0.46	0	0	0	6.89±1.02	0	0	0	17.43±1.04	0	0	0	10.86±1.26	0	0	0
	Luteolin 7-[6′'-(2-methylbutyryl)glucoside] I	94	Apigenin	0	10.15±0.49	0	0	0	20.63±2.07	0	0	0	21.23±1.81	0	0	0	BDT	0	0	0	11.98±0.37	0	0
	Unknown flavonoid glycoside III (apimaysin or flavone type)	96	Apigenin	0	0	0	0	0	0	0	0	BDT	0	0	0	0	0	0	0	6.73±1.45	0	0	0
	Luteolin 7-[6′'-(2-methylbutyryl)glucoside] II	97	Apigenin	0	15.87±1.33	0	0	0	46.26±2.92	0	0	0	47.45±0.88	0	0	0	7.88±1.02	0	0	0	20.06±0.89	0	0
	Unknown flavonoid glycoside VI (apimaysin or flavone type)	105	Apigenin	0	0	0	0	0	0	0	0	BDT	0	0	0	0	0	0	0	7.02±0.53	118.90±3.58	0	0
	Gardenin B	109	Apigenin	0	49.35±2.13	0	0	0	49.72±8.77	0	0	0	89.57±8.28	0	0	0	35.66±4.34	0	0	0	0.29±1.34	0	0
	Apigenin	110	Apigenin	0	0	0	0	16.11±0.67	BDT	3.09±0.22	0	0.98±0.66	BDT	0	0	6.30±0.91	9.29±1.46	0	0	BDT	BDT	0	0
	Thymusin 6-isobutyrate	113	Apigenin	0	14.57±0.61	0	0	0	BDT	0	0	0	BDT	0	0	BDT	0	0	0	0	0	0	0
*(continued on next page)*

**Table:** **Table 3***(continued)*


				Concentration of quantified compounds (µg/g)																			

Class	Name of Compound	Peak No	Standard used for quantification	W-B-F 1	W-B-B 2	W-K-F 3	W-K-B 4	RM-B-F 5	RM-B-B 6	RM-K-F 7	RM-K-B 8	RC-B-F 9	RC-B-B 10	RC-K-F 11	RC-K-B 12	BR-B-F 13	BR-B-B 14	BR-K-F 15	BR-K-B 16	BL-B-F 17	BL-B-B 18	BL-K-F 19	BL-K-B 20

Flavonol (FOL)	Quercetin 3,4′*-O-*di-beta-glucoside I	3	Quercetin 3*-O-*glucoside	61.07±0.97	0	0	0	23.24±1.54	0	0	0	37.81±1.52	0	6.72±1.28	0	27.57±1.89	0	5.04±0.30	0	42.26±1.97	0	6.25±0.33	0
	Quercetin 3,4′*-O-*di-beta-glucoside II	4	Quercetin 3*-O-*glucoside	5.58±0.64	0	0	0	0	0	0	0	4.12±0.26	0	0	0	BDT	0	0	0	0	0	0	0
	Taxifolin 3-glucopyranoside	11	Quercetin 3*-O-*glucoside	53.21±2.65	0	0	0	31.43±2.21	0	0	0	20.75±1.61	0	0	0	1993.21±14.55	0	160.42±6.88	0	78.71±2.98	0	0	0
	Taxifolin I	51	Quercetin 3*-O-*glucoside	0	0	0	0	0	0	0	0	0	0	0	0	1479.26±16.05	74.63±12.29	219.99±8.64	16.78±3.08	BDT	0	0	0
	3-Methylquercetin (isorhamnetin) I	52	Quercetin 3*-O-*glucoside	0	0	0	0	0	0	0	0	0	0	0	0	0	BDT	0	5.38±0.59	0	BDT	0	0
	Quercetin 3‐*O*‐glucoside (isoquercitrin)	62	Quercetin 3*-O-*glucoside	0	0	0	0	0	0	0	0	0	0	0	0	48.09±3.15	0	BDT	0	0	0	0	0
	Taxifolin II	70	Quercetin 3*-O-*glucoside	BDT	0	0	0	BDT	0	0	0	BDT	0	5.59±0.83	0	BDT	0	0	0	BDT	0	0	0
	Unknown flavonoid glycoside II (patuletin 7-galactoside or flavonol type)	84	Quercetin 3*-O-*glucoside	0	0	0	0	0	0	0	0	0	0	0	0	0	95.59±6.31	0	0	0	34.87±2.43	0	0
	Quercetin	99	Quercetin	BDT	0	0	0	0	0	0	0	0	0	0	0	BDT	84.63±10.36	0	0	BDT	BDT	0	0
	Kaempferol	100	Kaempferol	14.30±1.12	BDT	4.79±0.35	0	34.64±3.81	112.54±5.22	4.49±0.68	0	35.86±3.45	32.40±2.73	5.18±0.72	0	53.63±2.60	16.68±5.10	3.58±0.83	0	101.36±6.96	43.36±3.82	0	0
	3-Methylquercetin (isorhamnetin) II	102	Quercetin 3*-O-*glucoside	0	0	0	0	0	0	0	0	BDT	0	0	0	BDT	0	0	0	31.53±3.39	BDT	0	0

Proanthocyanidin (P)	Procyanidin B1	8	Procyanidin B1	0	0	0	0	BDT	0	0	0	0	0	0	0	1283.49±14.19	0	63.22±3.23	0	549.49±7.45	0	0	0
	Procyanidin I	36	Procyanidin B1	0	0	0	0	0	0	0	0	0	0	0	0	162.78±11.25	0	BDT	0	BDT	0	0	0

0 = not detected.

BDT = below the set UV-Vis detection threshold but confirmed by the mass spectrum.

UQ = unable to quantify due to large background noise/interference.

Peak number and 20 sorghum sample acronyms referring to Fig. 1.

W-B-F 1 = white colour Liberty sorghum, bran fraction, free form extract.

W-B-B 2 = white colour Liberty sorghum, bran fraction, bound form extract.

W-K-F 3 = white colour Liberty sorghum, kernel fraction, free form extract.

W-K-B 4 = white colour Liberty sorghum, kernel fraction, bound form extract.

RM-B-F 5 = red colour Mr-Buster sorghum, bran fraction, free form extract.

RM-B-B 6 = red colour Mr-Buster sorghum, bran fraction, bound form extract.

RM-K-F 7 = red colour Mr-Buster sorghum, kernel fraction, free form extract.

RM-K-B 8 = red colour Mr-Buster sorghum, kernel fraction, bound form extract.

RC-B-F 9 = red colour Nuseed Cracka sorghum, bran fraction, free form extract.

RC-B-B 10 = red colour Nuseed Cracka sorghum, bran fraction, bound form extract.

RC-K-F 11 = red colour Nuseed Cracka sorghum, kernel fraction, free form extract.

RC-K-B 12 = red colour Nuseed Cracka sorghum, kernel fraction, bound form extract.

BR-B-F 13 = brown colour IS131C sorghum, bran fraction, free form extract.

BR-B-B 14 = brown colour IS131C sorghum, bran fraction, bound form extract.

BR-K-F 15 = brown colour IS131C sorghum, kernel fraction, free form extract.

BR-K-B 16 = brown colour IS131C sorghum, kernel fraction, bound form extract.

BL-B-F 17 = black colour Shawaya Short Black 1 sorghum, bran fraction, free form extract.

BL-B-B 18 = black colour Shawaya Short Black 1 sorghum, bran fraction, bound form extract.

BL-K-F 19 = black colour Shawaya Short Black 1 sorghum, kernel fraction, free form extract.

BL-K-B 20 = black colour Shawaya Short Black 1 sorghum, kernel fraction, bound form extract.

## 2 Experimental Design, Materials and Methods

### 2.1 Chemicals and reagents

Standards of apigeninidin chloride, 7-methoxy-apigeninidin chloride and luteolinidin chloride were obtained from ChromaDex (Los Angeles, CA, USA). All other standards and chemicals were obtained from Sigma- Aldrich (Castle Hill, NSW, Australia). All chemicals used for the HPLC-DAD-ESI-QTOF-MS/MS and HPLC-DAD analyses were LC-MS grade.

### 2.2 Samples and preparation and phenolic extraction

Five different coloured sorghum grains were used. Liberty (White, W), Mr-Buster (Red, RM), Nuseed Cracka (Red, RC) sorghum grains were obtained from Nuseed Australia (Toowoomba, QLD, Australia) in 2019. IS131C (Brown, BR) and Shawaya Short Black 1 (Black, BL) sorghum grains were obtained from the experiment filed of Bentley campus of Curtin University, grown January to April 2019 (Bentley, WA, Australia). A TM05C SATAKE Testing Mill equipped with an #36 abrasive roller (SATAKE Corporation, Hiroshima, Japan) was used for grain decortication. Sorghum grains (200 g) were decorticated for 60 s to collect the bran fraction. The remaining grains were collected and further decorticated for 45 s to remove uncleared bran residues to give the kernel samples. Both bran and kernel fractions were ground by an EM0405 Multigrinder II grinder (Sunbeam, FL, USA), sieved 100% through a 500 µm brass sieve, and stored at –20 °C in vacuum bags in the dark before extraction.

The free and bound phenolic compounds were extracted according to previously published work [2]. For the extraction of free phenolic compounds, the ground sorghum sample (4 g) was mixed with 30 mL of 80% methanol solution under nitrogen gas, and the mixture was shaken at 25 °C and 150 rpm in the dark for 2 h. The mixture was centrifuged at 3500 g and 4 °C for 10 min to collect the supernatant, and the residue was re-extracted with 35 mL 80% methanol two more times. All supernatants were combined and evaporated to dryness by a rotary evaporator at 39–40 °C and 100 rpm for 10–15 min, and the resulting solid was re-dissolved in 20 mL of 100% methanol and stored under nitrogen gas at −20 °C in the dark for 1–3 day until analysis. For the extraction of free phenolic compounds, the residue remaining after the free phenolic extraction was mixed with 30 mL of 2 M HCl under nitrogen gas and heated at 100 °C for 60 min for hydrolysis. Then, 40 mL ethyl acetate was added and mixed thoroughly and wait for about 5 min for partition. After partitioning, the ethyl acetate fraction was collected, and the hydrolysate was re-extracted with 50 mL ethyl acetate five more times. All ethyl acetate fractions were pooled and evaporated to dryness by a rotary evaporator at 39–40 °C and 100 rpm for 10–15 min, and the resulting solid was re-dissolved in 20 mL of 100% methanol and stored under nitrogen gas at −20 °C in the dark for 1–3 day until analysis.

### 2.3 HPLC-DAD-ESI-QTOF-MS/MS qualitative analysis

The identification of phenolic compounds was performed by an Agilent 1200 series HPLC system, equipped with a vacuum degasser, auto-sampler, binary pump and diode-array detection (DAD), and coupled with an Agilent 6520I Accurate-Mass Q-TOF LC/MS (Agilent Technologies, Santa Clara, CA, USA). Chromatographic separation was achieved on a reverse phase Synergi Hydro-RP 80A LC column (4 µm, 250 × 4.6 mm) protected by an AQ C18 guard column (4.0 × 3.0 mm) (Phenomenex, Lane Cove, NSW, Australia).

The HPLC-DAD-ESI-QTOF-MS/MS (and also the HPLC-DAD in Section 2.4) analysis was based on previously published work [3,4], with modifications and optimisation. The LC and MS conditions, mobile phases, and elution program were optimised for maximum peak separation and signal intensity and quality. The LC conditions: column temperature 30 °C, injection volume 10 μL. DAD settings: scan range 190–720 nm at 2.0 nm step, and monitoring wavelength at 280 nm for hydroxybenzoic acid, flavan-3-ol and flavanone, 320 nm for hydroxycinnamic acid, 340 nm for flavone, 370 nm for flavonol and 485 nm for 3-deoxyanthocyanidin. The mobile phase A was 1.0% formic acid in milli-Q water and mobile phase B was LC-MS grade acetonitrile. The flow rate was 0.650 mL/min, with an 80 min elution program was set as follows: 5% B (0 min), 5–8% B (5 min), 8–21% B (30 min), 21–35% B (19 min), 35–60% B (9 min), 60–100% B (4 min), 100% B (5 min), 100–5% B (0.1 min), 5% B (7.9 min). For MS analysis, negative mode via a dual electrospray ionisation source (ESI) was employed. The MS acquisition parameters are as follows: drying gas N_2_, temperature 325 °C, gas flow 9 L/min, nebuliser 45 psi; capillary voltage 3500 V, fragmentor 175 V; MS scan range 90–1000 m/z. The MS/MS was performed in auto mode with MS/MS scan range 90–850 m/z and collision energy 15–30 eV.

The data was analysed by MassHunter Qualitative software (Agilent Technologies, Santa Clara, CA, USA). The integration thresholds were set as peak area > 30000 counts for UV–Vis chromatogram and > 1 counts for MS chromatogram, and only the MS and UV-Vis matched peaks, i.e. peaks that are present in both MS and UV-Vis chromatograms with the peak area above the thresholds, were selected for further analysis. Compound identification and characterisation were based on comparing the retention time, UV-Vis, MS and MS/MS spectra with authentic standards, database, and published literature as follows:

(1) Standards: a total of 27 standards were used for identification, of which 15 matching compounds were identified in the tested sorghum samples, as shown in Table 1.
(2) Published literature: some compounds were identified by comparing their data profile with that reported in published literature (of sorghum studies), and these compounds were double checked by database for verification in the following step.
(3) Database: MS-DIAL 4.0 coupled with MS-FINDER 3.24 software using MSMS-Public-Neg-VS14 database was the main tool used for identification [5,6]. The settings were MS-DIAL score > 80 and MS-FINDER score > 7.5, and compounds/peaks below these scores were not selected for identification. Besides, the UV-Vis spectrum of each compound was used to assign it to a subclass according to its specific UV-Vis absorption/peak pattern [7], and compounds without matched subclass UV-Vis absorption/peak pattern were not selected for identification. Also, online UV–Vis (SpectraBase) and Mass (ChemSpider, Phenol-Explorer and MassBank) database were used for double verification when available.
(4) Mass error: only compounds with mass error ≤ ±10 ppm, and compounds with mass error > ±11 ppm but identified by standards or having a high MS-DIAL score > 90, were selected for identification and verification.

### 2.4 HPLC-DAD quantitative analysis

The quantification of phenolic compounds was performed by an Agilent 1260 series HPLC system equipped with a DAD (Agilent Technologies, Santa Clara, CA, USA), and the same column, mobile phase and conditions were applied as described above in Section 2.3. The data was intergraded by Agilent OpenLAB Workstation software (Agilent Technologies, Santa Clara, CA, USA), and the integration threshold was set as peak area > 1. Compounds with standards were directly quantified by the standards, and compounds without available standards were semi-quantified by selecting structurally similar standards or the standards of the same subclass based on their functional group and chemical structure (i.e. core structure and functional group), as shown in Table 3. Compounds without structurally matched standards were not quantified. The calibration curves of standards were created at their specific monitoring wavelengths as described above in Section 2.3, and compounds were quantified/semi-quantified at their selected monitoring wavelengths. The semi-quantification was performed on the basis of that phenolic compounds of the same subclass with similar core structure and functional group have similar UV-Vis absorption pattern/peaks at 200–600 nm [5], and this method has been used in many studies [8], [9], [10].

The quantification method was validated for linearity, limit of detection (LOD), limit of quantification (LOQ) and precision (repeatability). Calibration curves were obtained at eight levels of concentration of standards, except for procyanidin (seven levels of concentration). Method linearity was tested on the basis of calibration curves, which were processed using linear regression. LOD and LOQ were calculated based on the standard deviation of the regression line (SD) and the slope (S) according to the formulae: LOD = 3.3(SD/S) and LOQ = 10(SD/S). Precision (repeatability) was evaluated by analysing three replicates (consecutive injections) of three different concentrations of standards according to Table 2, and the relative standard deviation (RSD) at each concentration of standard was calculated. All the calibration and method validation parameters for the quantification of phenolic compounds were presented in Table 2. The experiment was carried out in triplicate and data were expressed as mean ± standard deviation.

## Declaration of Competing Interest

The authors declare that they have no known competing financial interests or personal relationships which have, or could be perceived to have, influenced the work reported in this article.
